# Recent Clinical and Preclinical Advances in External Stimuli-Responsive Therapies for Head and Neck Squamous Cell Carcinoma

**DOI:** 10.3390/jcm12010173

**Published:** 2022-12-26

**Authors:** Zheng Jiang, Xin Yang, Mailudan Ainiwaer, Fei Chen, Jun Liu

**Affiliations:** Department of Otolaryngology, Head and Neck Surgery, West China Hospital, Chengdu 610041, China

**Keywords:** stimuli-responsive, photodynamic, photothermal, sonodynamic, radiodynamic, microwave dynamic, microwave thermodynamic, magnetothermal, magnetodynamic, nanotechnology

## Abstract

Head and neck squamous cell carcinoma (HNSCC) has long been one of the most prevalent cancers worldwide; even though treatments such as surgery, chemotherapy, radiotherapy and immunotherapy have been proven to benefit the patients and prolong their survival time, the overall five-year survival rate is still below 50%. Hence, the development of new therapies for better patient management is an urgent need. External stimuli-responsive therapies are emerging therapies with promising antitumor effects; therapies such as photodynamic (PDT) and photothermal therapies (PTT) have been tested clinically in late-stage HNSCC patients and have achieved promising outcomes, while the clinical translation of sonodynamic therapy (SDT), radiodynamic therapy (RDT), microwave dynamic/thermodynamic therapy, and magnetothermal/magnetodynamic therapy (MDT/MTT) still lag behind. In terms of preclinical studies, PDT and PTT are also the most extensively studied therapies. The designing of nanoparticles and combinatorial therapies of PDT and PTT can be referenced in designing other stimuli-responsive therapies in order to achieve better antitumor effects as well as less toxicity. In this review, we consolidate the advancements and limitations of various external stimuli-responsive therapies, as well as critically discuss the prospects of this type of therapies in HNSCC treatments.

## 1. Introduction

Head and neck squamous cell carcinoma (HNSCC), which accounts for approximately 90% of head and neck cancers, has long been one of the most prevalent cancers worldwide [1]. Associated with high malignancy and poor prognosis, the HNSCC can affect the oral cavity, nasal cavity, sinuses, pharynx and larynx [2]. Current mainstay treatments for HNSCC include surgery, chemotherapy, radiotherapy and immunotherapy, which all carry some disadvantages. For example, surgical excision can be a potential stimulator that triggers the local invasion or distant metastasis of the tumor [3]; chemotherapies can lead to hepatotoxicity, nephrotoxicity, gastrointestinal disturbance, bone marrow suppression or even carcinogenesis [4,5]; radiotherapy can result in dysphagia, xerostomia and osteoradionecrosis of the jaw, which can negatively influence patients’ quality of life [6,7]; in terms of immunotherapy, though it looks promising and has a good synergistic effect with chemotherapy, some patients still experienced hyperprogression while receiving the anti-PD-1/PD-L1 treatment [8]. Despite receiving comprehensive treatments, about 65% of the patients experience tumor recurrence or metastasis, while most of them are considered incurable given palliative chemotherapies [2,9], and the overall five-year survival rate of HNSCC is still unsatisfactory with a percentage of 40–50% [10]. Hence, novel treatments with better tumor-controlling potency as well as safety are in urgent need, and thanks to the innovations of nanotechnology, we now possess many more weapons in our ‘antitumor toolkit’.

**Figure 1:**
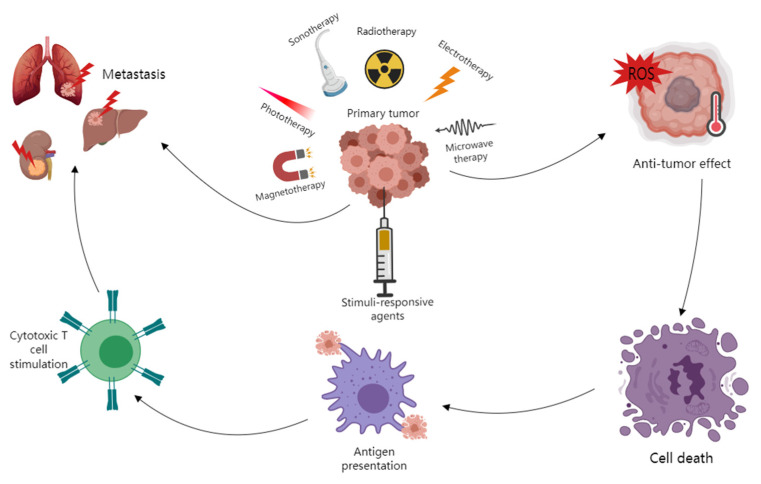
Demonstration of external stimuli-responsive therapies. External stimuli such as light, radiation, microwave, alternative magnetic field (AMF) and ultrasound (US) induce the generation of heat or reactive oxygen species (ROS), thus resulting in tumor death. Antigens are released upon tumor death and captured by antigen-presenting cells, which further trigger cytotoxic immune cell activation against metastatic tumors.

Nanotechnology provides us with various direct and indirect methods of treating cancer. For example, a nanodelivery system can carry anticancer drugs directly to the cancer lesion and reduce the blood drug concentration, thus minimizing the toxicity of anticancer drugs; nanomaterials that can be excited by a stimulator (light, sound, radiation, etc.) can kill cancer cells directly through producing reactive oxygen species (ROS) or energy (radiation and heat) (Figure 1); some nanomaterials work as immunomodulators that trigger immune reactions regionally or systematically and cause indirect anticancer effects [10,11,12].

Several works have described the applications of nanotechnology in HNSCC, but a review going through all types of potent stimuli-responsive nanotherapies is still in urgent need [2,9,10,11,12]. Accordingly, this review summarizes and introduces mechanisms of various stimuli-responsive nanotechnology-based therapies such as photodynamic therapy (PDT), photothermal therapy (PTT), sonodynamic therapy (SDT), radiodynamic therapy (RDT), microwave dynamic/thermodynamic therapy and magnetothermal/magnetodynamic therapy (MDT/MTT), electrodynamic therapy (EDT) as well as their preclinical or clinical development status. This review also introduces some novel nanotechnology-based therapies that have not been studied in HNSCC in order to guide future research in this field.

## 2. Photodynamic Therapy (PDT)

### 2.1. Introduction to Photodynamic Therapy

PDT has been improved greatly since 1898, when Oscar Raab first established the basic concept of photodynamic treatment [13]. PDT involves the systematic or local application of a special photosensitizer, which is excited by illumination with visible light of an appropriate wavelength; the excited photosensitizer can generate reactive oxygen species (ROS), thus damaging the adjacent biomolecules such as lipids, proteins or nucleic acids.

### 2.2. Introduction to Sensitizers

Some photosensitizers such as Foscan have already been approved for palliative treatment and have shown robust clinical benefits in HNSCC patients [14,15]. Photosensitizers can be divided into two categories—porphyrin or non-porphyrin compounds; the first clinically approved photosensitizers are hematoporphyrins (HpD), which are still being widely used in clinical settings, but the skin toxicity of HpD is concerning physicians; efforts to reduce the toxicity and to maximize the concentration in the tumor led to the development of second-generation photosensitizers such as texafirins (Lutrin), phenylporphyrins (m-THPP), pyropheophorbide (HPPH), aminolevulinic acid (5-ALA), chlorins (mTHPC, talaporphin, Ce6), bacteriochlorins (Tookad, redaporphin) and porphyrazines (Photosens) [16]. In order to further improve the selective enrichment of the photosensitizers, researchers proposed the concept of the third-generation photosensitizer, which is characterized by combining with a targeting vehicle for direct delivery to the tumor, thus maximizing the antitumor effect while minimizing the systemic adverse effect.

### 2.3. Clinical Development of Photodynamic Therapy

Porphyrin sodium [17,18], mTHPC [15,19] and 5-ALA [20,21] are popular photosensitizers that have been extensively studied both preclinically and clinically; those photosensitizers all showed potent antitumor effects in HNSCC patients of various TNM stages. Chlorin derivatives such as HPPH [22,23] and talaporphin [24] are relatively newer and less studied in HNSCC, but they also have shown good clinical outcomes as well as low incidence of phototoxicity [22,25]. So far, there has been only one clinical study investigating the use of talaporphin in HNSCC patients with different TNM stages; the outcome of talaporphin treatment in this study was prominent, with a complete response of 75%, but the result needs to be further examined and confirmed in large clinical trials due to the fact that the sample size was too small in this study [24].

### 2.4. Preclinical Development of Photodynamic Therapy

The recent trend in preclinical photodynamic therapy development mainly lies in two fields; one is by modifying the biomaterials or combining them with other materials in order to improve their tumor affinity or to enhance the antitumor effect, and the other one is to modify the tumor microenvironment and supply oxygen for ROS generation.

A recent study on phthalocyanine photosensitizer IR700DX showed that the conjugation between the photosensitizer and EGFR-targeting antibody cetuximab can significantly improve the tumoral tissue accumulation of the photosensitizer [26]. The conjugated IR700DX also showed significantly improved long-term tumor control in a mouse EGFR-overexpressing human head/neck OSC-19-luc2-cGFP tumor model, which indicated that the targeting drug conjugation can be an effective way of improving the specific delivery of photosensitizers in the future PDT development for HNSCC. Efforts have also been made in potentiating the PDT effect inside the tumor by creating a more favorable microenvironment for photosensitizers. For instance, Tao et al. encapsulated Ce6 together with the hypoxia regulator resveratrol into a small-sized micelle with EGFR targeting ligand GE11; the resveratrol inhibits cellular oxygen consumption, thus providing sufficient oxygen for PDT [27]. Such a combination showed superior antitumor effects in an orthotopic oral squamous cell carcinoma model and is inspiring for future nanoparticle designs. Another interesting nanoparticle design in glioblastoma treatment, which offers new insight into developing dark PDT (dPDT: PDT without external light stimulation) for HNSCC. Lu et al. combined Ce6 with lactate oxidase (LOX), hemoglobin (Hb) and Bis 2,4,5-Trichlorophenyl-6-Carbopentoxyphenyl Oxalate (CPPO). LOX converts the lactate, which is the tumor metabolite, into pyruvic acid and H_2_O_2_, the H_2_O_2_ reacts with the CPPO, thus releasing energy and exciting the Ce6, and the hemoglobin works as an oxygen donor for both lactate catabolism and PDT. All four materials are assembled into nanoparticles made from U251 glioma cells for specialized delivery to the tumor [28]. Such a synergistic system demonstrated a strong therapeutic effect in animal models, the success of this design offers new insights into tumor metabolite utility, chemiexcited PDT and delivery particle design. Moreover, perfluocarbon [29], MnO_2_ [30] and hemoglobin-based nanostructures [31] have been explored in altering the hypoxic tumor microenvironment and all achieved ideal effects to a certain extent in various types of cancer.

Nanotechnology-based drug delivery systems are a research hotspot in recent years; they can carry and deliver anticancer drugs directly into tumors, thus enhancing the antitumor effect. The most commonly used active targeting ligands include transferrin [32], folic acid [33] and Arg-Gly-Asp [34], but none of them showed specific affinity towards HNSCC [35]. Song et al. used a novel approach by combining Ce6 with polyethylene glycol diamine (PEG) and integrated them into the shell of the nanoparticles that encapsulate cisplatin and metformin. Laser stimulation leads to the deformity of the shell, thus releasing the drugs in situ. The PDT and PTT triggered by lasers showed synergistic effects with the chemotherapy; additionally, this combination therapy showed significantly lower systemic toxicity than free cisplatin [35]. The same study design can be repeated using different chemotherapy combinations or probably immunotherapy medications.

## 3. Photothermal Therapy (PTT)

### 3.1. Introduction to Photothermal Therapy

PTT was first used by Goldman in 1966 by ablating a melanoma with heat generated by laser [36]. The PTT in modern days uses photothermal conversion agents (PTAs) to generate heat under near-infrared (NIR) light irradiation to ablate tumor cells [36]. It was reported that a temperature of 41 °C can cause vasodilation, thus increasing the blood perfusion to the tumor as well as causing heat shock response of cells; temperatures above 46 °C can lead to irreversible cell death [37].

### 3.2. The Current Clinical Development of Photothermal Therapy

However, disappointingly, the clinical development status of PTT is considerably behind that of PDT even though it has shown great potential in preclinical tumor models [36]. There is only one PTT clinical trial concerning head and neck cancer that did not demonstrate preferable results; in it, among 11 enrolled patients, 3 of them died within 6 months, and 6 of them did not complete the entire treatment (NCT00848042).

### 3.3. Introduction to Sensitizers

In the study of HNSCC, the most commonly used PTT agents were noble metals such as Au [38], Ag [39], Pd [40] and Pt [41], and they were usually combined with certain biomaterials such as polyethylene glycol (PEG) to increase their water solubility and to reduce their immunogenicity in vivo [42]. Au nanoparticles are one of the most explored and promising PTT agents among noble metals due to their outstanding photothermal conversion [43]; the Au nanoparticles used in PTT have different morphologies including nanorods, nanospheres, nanostars and nanoflowers [12]. Carbon-based nanomaterials carry better biocompatibility than metal-based nanomaterials, but they have relatively poorer NIR light absorption ability and water solubility. Morphologies of carbon-based PTT agents are mainly graphene [44] or carbon nanotubes [45]. Other materials such as metal compounds [46,47,48] and organic nanoparticles [49,50] have also been extensively studied preclinically in HNSCC cell models and have shown promising efficacy.

### 3.4. Preclinical Development of Photothermal Therapy

Efforts have been made in improving the innate disadvantages of each type of PTT agent. In noble metal nanomaterial agents, it is challenging to deliver them specifically to the tumor tissue while ensuring efficient biodegradation and biosafety. Various tumor-targeting coatings have been fabricated on nanomaterials to facilitate the accumulation in tumor tissues and cells. Sun et al. coated gold nanorods with a cancer cell membrane (GNR@Mem), which showed preferable homotypic targeting to cancer cells in vitro [51]. The in vivo study also showed a preferable accumulation of gold nanorods inside the tumor, which were mostly excreted via feces and urine three days after injection. Another way of improving the noble metal nanoparticles is to conjugate them with targeting agents. Melancon et al. conjugated gold nanoshells with an anti-EGFR monoclonal antibody C225 (cetuximab); C225-SPIO@Au NS showed strong selective accumulation in EGFR-positive SCC cell lines both in vitro and in vivo, and the selective accumulation of PTT agents also potentiated a therapeutic effect in subsequent experiments [52].

Concerning carbon-based nanomaterials, the photothermal conversion ability and water dispersibility have long been concerning the researchers; an efficient method of increasing the overall photothermal conversion ability is to conjugate or combine it with other materials with photothermal ability. For example, Wang et al. used single-walled carbon nanotubes to encapsulate hyaluronic acid-5β-cholanic acid nanoparticles-bound indocyanine green (IHANPT); they exhibited superior synergistic photothermal effect and showed good outcomes in SCC7 cell line animal models, tumors were mostly ablated and no recurrence was observed in the IHANPT group. Such a combination also showed good selective delivery due to the CD44-targeting behavior of hyaluronic acid-5β-cholanic acid nanoparticles [45]. Graphene and its derivative have been attracting people’s attention in biomedicine research due to its special surface properties, excellent photo-thermal conversion efficiency and the potential for extra engineering. Conjugating graphene with other materials with stronger photothermal conversion ability is the method of choice to achieve the ideal photothermal effect in preclinical studies. Gao et al. seeded gold into graphene to achieve a synergistic effect [44], while Shakerian Ardakani et al. further investigated the combinational effect of PTT and radiodynamic therapy (RDT) by using Fe_3_O_4_@Au/reduced graphene oxide nanostructures as the photo- and radiosensitizers [53]. Graphene-based photosensitizers all showed good in-vivo antitumor effects as well as good biocompatibility in healthy cells. The water dispersibility of carbon-based materials is currently being addressed by chemists; some carbon materials with good water dispersibility have already been developed and may have the potential of being used in future photothermal conversion agent development [54].

Metal compounds have also attracted much attention due to their good biocompatibility, high photothermal conversion effect, low cost, good photothermal stability and low cytotoxicity. Iron, copper and molybdenum are the most-used metal substrates in PTT agent development. Fe_3_O_4_ nanoparticles can induce hyperthermia under NIR laser radiation due to their unique magnetism; efforts have been made in modifying the serum dispersibility and improving the cellular uptake, thus minimizing the cytotoxicity [46]. Copper sulfide (Cu-S) nanomaterials are one of the most promising copper-base agents with strong photothermal conversion, low cytotoxicity and low cost [55]. The main ideas of modifying copper sulfide agents are to either conjugate them to target nanomaterials in order to facilitate specific delivery [56] or to combine them with certain materials and study the combinational therapy [57]. Molybdenum (Mo) is an emerging metal that carries great potential in PTT agent development. Qian et al. found that MoP_2_ nanorods can achieve an ideal photothermal effect in vivo and can enhance chemodynamic therapy [58]. Chen et al. synthesized chiral molybdenum (Cys-MoO_3_-x) nanoparticles, which was proven to have low cytotoxicity and showed a good PTT effect in OSCC treatment [48]. Despite the excellent photothermal conversion ability and biocompatibility, the insolubility is limiting the use of molybdenum compounds, which requires further research; moreover, targeted delivery of molybdenum-based PTT agents should also be further explored in future research [59].

In terms of organic nanoparticles, their advantages are excellent biocompatibility and biodegradability, which have overcome one of the biggest obstacles that is keeping PTT from clinical use. Though this seems promising, the rapid degradation is limiting their photothermal conversion ability, thus undermining their therapeutic effect. Thus, the majority of studies on organic PTAs use combinational therapies to achieve better treatment effects in HNSCC. NIR dyes are the most extensively studied organic PTAs. Dyes such as indocyanine green possesses both photodynamic and photothermal effects, which exhibited synergistic antitumor effects in experiments [49]. NIR dyes also showed good synergistic effects with doxorubicin, cisplatin or docetaxel chemotherapy [12,60,61,62,63]. Another type of organic PTA is conductive polymers such as polypurrole and hydroxyapatite, which also carry preferable photothermal conversion efficiency and have been proven to possess good synergistic effects with doxorubicin chemotherapy [64,65,66].

There is another disadvantage limiting the efficacy of PTT—the physical limitation of the light penetration depth. The traditional NIR used by PTT usually has a skin penetration of less than 1 cm, leaving the deep-tissue tumor unaffected. Thus, NIR-II laser-responsive PTAs need to be paid more attention to in order to achieve better therapeutic effects in deep-situated tumors [67].

So far, there have not been many published clinical studies on PTT due to the long-term biological behavior of metal-based nanomaterials despite their outstanding antitumor efficacy. The difficulty in biodegradation will lead to accumulation in organs and cause potential toxicity. Thus, further investigation should be conducted concerning biodistribution, pharmacokinetics and toxicity as well as biodegradation in order to facilitate the future clinical use of PTT.

## 4. Sonodynamic Therapy (SDT)

### 4.1. Introduction to Sonodynamic Therapy

SDT was first derived from PDT by Yumita et al. in 1989, who found that several hematoporphyrin derivatives can also be activated by ultrasound, thus causing cell damage [68]. The general mechanism of SDT is that when excited by low-intensity ultrasound, the sonosensitizer generates ROS from the molecular oxygen, thus initiating cell death; other than that, SDT was also indicated to have an inhibitory effect on cancer growth with an unknown mechanism [69,70].

### 4.2. Introduction to Sensitizers

The categories of sonosensitizers include porphyrin-based, xanthene-based, non-steroidal anti-inflammatory drug-based and other sonosensitizers. Among those, the porphyrin-based sonosensitizers (HMME [71], PpIX [72], Ce6 [73]) are the most extensively studied agents due to their good biocompatibility; they also have a good PDT effect, which can work synergistically with SDT in antitumor therapy. Xanthene-based sonosensitizers (Erythrosin B, rose bengal) are featured with very high sonodynamic efficiency under ultrasound, but some disadvantages that are concerning the researchers are their low accumulation in tumor tissues, rapid sequestration in the liver and subsequent clearance [74]. Non-steroidal anti-inflammatory drugs such as tenoxicam [75] and piroxicam [76] can have a strong sonodynamic effect under ultrasound stimulation and exhibited preferable antitumor effects in preclinical studies. Other than the agents mentioned above, there are also some less-studied sonosensitizer candidates such as some traditional photosensitizers (curcumin [77], indocyanine green [78], hypocrellin B [79] and 5-ALA [80]) or some metal-based nanoparticles such as TiO_2_ nanoparticles [81] and SiO_2_ nanoparticles [82].

### 4.3. The Current Clinical Development of Sonodynamic Therapy

So far, there have not been any clinical studies on SDT in HNSCC patients yet, and only two human subject studies using SDT were identified in an extensive literature search. Wang et al. used the combinational therapy of SDT and PDT (Sonoflora 1 as the agent) for metastatic breast carcinoma treatment in three patients and all three patients showed partial or complete responses [83]. Kenyon et al. used Sonnelux 1 SonneMed, LLC, Winchester, MA, USA, as the sonosensitizer and photosensitizer to treat 115 cancer patients with advanced metastatic states, and the median survival time was extended for most of the patients according to the report, which is very encouraging [84]. Clinical studies of SDT in HNSCC patients are encouraged since there are some sonodynamic agents such as 5-ALA [20,21] that have already been extensively tested in human subjects and have shown good biocompatibility and biosafety; combinational therapy of SDT and PDT can be conducted in HNSCC patients with deep tissue involvement that is limiting the efficacy of phototherapy alone.

### 4.4. Preclinical Development of Sonodynamic Therapy

Protoporphyrin IX is the most extensively investigated sonosensitizer in HNSCC. Lv et al. discovered an ideal antitumor effect of PpIX-based SDT in SAS cell lines both in vitro and in vivo; they also concluded that the PpIX-based SDT has the potential of inducing G2/M phase arrest as well as apoptosis of SAS cells [85]. 5-ALA-based sonosensitizer also showed great potential in inducing apoptosis in SAS cell lines according to some recent preclinical studies [86,87]. A study on HMME-based SDT showed that the therapy decreased the tumor cell survival rate by 27% and the apoptotic cells were significantly increased in the SDT treatment group [88]. TiO_2_-based sonosensitizer activated by high-intensity focused ultrasound (HIFU) also demonstrated a preferable effect in HSC-2 cell line models. Some more recent studies focused on developing new SDT agents or synthesizing new nanoparticles that can enhance the SDT effect. Pourhajibagher et al. found that nano emodin transfersome (NET) has the potential of generating ROS as well as inducing apoptosis in HNSCC cell lines [89]. Sun et al. combined sulfide dioxide (SO_2_) and 5-ALA together and then co-assembled them with methoxyl poly(ethylene glycol)-b-poly(l-lysine) (mPEG-b-PLL) in order to consume the overproduced glutathione in the tumor microenvironment, thus enhancing ROS generation; such an SDT therapy showed preferable antitumor effects in both melanoma and squamous cell carcinoma in mouse models [90]. The use of sonodynamic therapy in HNSCC is relatively less-studied, unlike that in photodynamic or photothermal therapies; future studies can pay more attention to combinational treatments such as SDT + PTT [56], SDT + PDT [91] and SDT + chemotherapy [92]. Additionally, novel nanoparticle designs from PDT and PTT research can be referenced in developing more potent SDT agents in order to achieve better tumor affinity or a more favorable tumor microenvironment, thus enhancing SDT efficacy [93,94].

## 5. Radiodynamic Therapy (RDT)

### 5.1. Introduction to Radiodynamic Therapy

RDT, also known as X-ray photodynamic therapy, was first introduced in the mid-1950s [95]. RDT is based on X-ray-induced excitation of special X-ray-sensitive photosensitizers or UV-vis-emitting radioluminophores/quantum dots/semiconductors coupled with photosensitizers [95]. The antitumor mechanism of RDT is similar to that of PDT, as it also generates ROS to damage the cancer cells, but RDT is superior to PDT in certain types of tumors that involve deep tissues, since the X-rays can efficiently penetrate and reach the deep-situated tumor tissue. X-ray photosensitizers can be roughly classified into three major categories—rare-earth element-based sensitizers (Tb [96], Gd [96], Ce [97], La [98], Eu [99]), transition metal-based sensitizers (Zn [100]) and other metal-based sensitizers (Au [101], Ti [102], Hf [103]).

### 5.2. Introduction to Sensitizers

So far, there is no clinical application or study on radiodynamic therapy in HNSCC; in addition, preclinical study on RDT application in HNSCC cell lines is still lacking. Nanoparticles from previous studies on other types of cancer can be referenced to develop RDT designs for HNSCC treatment. The most basic design of the nanoparticles includes scintillating particles combined with photosensitizers; for example, Zou et al. synthesized the LaF_3_:Ce(3+)/DMSO nanoparticle and combined it with PpIX and achieved preferable antitumor effects in prostate cancer models [104]. Novel radiosensitizers can be designed by attempting either different combinations or utilizing other metals with X-ray absorption ability. For example, a very recent study carried out by Ni et al. proposed a bismuth-based metal-organic framework as a new radiosensitizer that carries great antitumor potential in pancreatic and prostate cancer models and showed a preferable synergistic effect with immunotherapy [105].

### 5.3. Preclinical Development of Sonodynamic Therapy

To improve the accumulation of the radiosensitizers in the tumor tissue, the nanoparticles can be conjugated to a certain targeting moiety to facilitate specific delivery [106]. Some novel nanocarrier designs in PDT studies can also be referenced to synthesize nanoparticles with preferable tumoral accumulation, thus enhancing the antitumor effect [27]. Other than the specific delivery, the modification of the hypoxic tumor microenvironment can also effectively enhance the antitumor effect of radiosensitizers since they also require oxygen molecules to generate ROS. By combining Hemoglobin [31] or H_2_O_2_ catalysts such as MnO_2_ [30] into the nanoparticle, the ROS generation in the tumor region is likely to be increased, thus enhancing the antitumor effect.

## 6. Microwave Dynamic and Microwave Thermodynamic Therapy

### 6.1. Introduction to Microwave Therapy

Microwaves have long been utilized in clinical settings for tumor ablation, which is known as microwave thermal therapy. The mechanism of its antitumor effect is similar to that of photothermal therapy; while a temperature of 41 °C can cause vasodilation, thus increasing the blood perfusion to the tumor as well as causing heat shock response of cells, temperatures above 46 °C can lead to irreversible cell death [37]. Various sensitizers have been developed to enhance the thermogenesis of microwave treatment [107,108], and enhanced microwave thermal therapy was subsequently named ‘microwave thermodynamic therapy’ [109]. Some microwave heating sensitizers such as ionic liquid have the ability to generate ROS under stimulation, which is known as ‘microwave dynamic therapy’ [110].

After an extensive literature search, we failed to find any studies on HNSCC both clinically and preclinically. Nanoparticle designs for other types of cancer can be referenced and adapted for HNSCC treatment development. Here, we introduce some representative nanoparticle designs that showed great potential in other cell lines.

### 6.2. Introduction to Sensitizers and the Preclinical Development of Microwave-Based Therapies

Wu et al. synthesized the zeolitic imidazolate Frameworks-8 (ZIF-8) and coated it with bovine serum protein, and the BSA@ZIF-8 exhibited favorable microwave thermal conversion and demonstrated a preferable antitumor effect in H22 xenograft models [107]. Chen et al. encapsulated microwave-sensitive ionic liquid with zirconia (ZrO_2_) nanoparticles that were co-decorated with mitochondrial-targeting molecules of triphenylphosphonium (TPP) and tumor cell-targeting peptide iRGD; the nanoparticle complex showed an ideal targeting effect and sufficient accumulation in tumor tissue, and thus achieved preferable antitumor effects in an H22 tumor model [108]. A very recent study carried out by Zhu et al. used a brand-new nanoparticle design; they synthesized a Ca^2+^-surplus alginate hydrogel, which demonstrated preferable microwave-thermal conversion and the immunostimulatory effect. Such a hydrogel also carries favorable biocompatibility, which gives it great potential in future clinical translation [111]. This study also suggests to us that in situ-formed metallo-alginate hydrogel would have great potential as a microwave sensitizer and immunostimulatory agent in treating various cancers including HNSCC; metallo-alginate hydrogel can be further designed or modified to better potentiate the microwave-thermal conversion as well as improving the focal accumulation.

## 7. Magnetothermal and Magnetodynamic Therapy

### 7.1. Introduction to Magnetic Therapy

Magnetothermal therapy is a hyperthermia therapy mediated by an alternating magnetic field (AMF) and magnetic nanoparticles. The alternating current magnetic fields activate the nanoparticles and produce heat via magnetic hysteresis losses or Néel relaxation [112]. There is another type of magnetic therapy named ‘magnetothermaodynamic therapy’ that generates heat and ROS, thus producing a combinatorial antitumor effect [113]. Iron oxide and ferrite are the most commonly used substrates in magnetic nanoparticle synthesis, and colossal magneto-resistive materials such as manganese-based perovskite oxides are relatively less used but they also have good magnetothermal conversion ability and carry great potential in future biomedical applications [114].

### 7.2. Introduction to Sensitizers and Preclinical Development of Magnetic Therapies

Though hyperthermia treatment has long been used for HNSCC treatment [115], magnetothermal or magnetodynamic therapy have not been applied clinically yet, and several preclinical researches on HNSCC all showed good responses. Su et al. used superparamagnetic iron oxide nanoparticles as the magnetothermal agents and modified them with anti-CD44 antibodies for targeted delivery (CD44-SPIONPs). The CD44-SPIONPs exhibited good biocompatibility and good inhibitory effect in the cancer stem cells of Cal-27 cells (human oral squamous cell carcinoma) [116]. Legge et al. used a similar design, in which the iron oxide nanoparticles were encapsulated into a silica coating in order to improve the biocompatibility, and the complex was subsequently conjugated to an antibody targeting αvβ6 integrin to ensure specific delivery. The magnetothermal therapy showed good cell killing in cultured VB6 cell lines [117]. Tsai et al. also utilized targeting antibodies to improve the targeted delivery of the magnetothermal agents, and the MMP-1-FeAu nanoparticles conjugate triggered 89% HSC-3 cellular death [118].

In future studies of magnetic therapies for HNSCC, colossal magneto-resistive materials can probably be attempted due to their high magnetic transition temperature, TC (≥360 K) [119]. Magnetodynamic therapy can also be tested for its efficacy in HNSCC since no relevant studies were found in HNSCC treatment [113,120]. Efforts in improving biocompatibility and targeted delivery as well as reducing cytotoxicity should be made in future studies.

## 8. Electrodynamic Therapy (EDT)

### 8.1. Introduction to Electrodynamic Therapy and Sensitizer

Electrodynamic therapy has a similar mechanism as other dynamic therapies; the main mechanism is the generation of reactive oxygen species (ROS) using platinum nanoparticles (PtNP) under the stimulation of an alternating electric field. The ROS subsequently triggers cell apoptosis and immune reactions [121]. Unlike PDT or SDT, the mechanism of ROS production is completely different in EDT; it does not rely on O_2_ or H_2_O_2_ in the tumor microenvironment to generate cytotoxic hydroxyl radicals. Instead, it decomposes water and generates ROS with the assistance of chlorine ions [122]. Moreover, owing to the physical property of electricity, the EDT is able to ablate tumors with relatively larger dimensions, which makes it a therapy with great potential in treating various solid tumors.

### 8.2. Preclinical Development of Electrodynamic Therapy

So far, there has not been much research on EDT, and its clinical development is also preliminary. In terms of HNSCC, we did not find any preclinical or clinical studies on it even after an extensive literature search. Within all of the available research papers, BALB/c mice bearing 4T1 tumors are the most frequently studied animal model. Gu et al. tested the antitumor efficacy of PtNP in 4T1 tumor cells and achieved preferable antitumor effects both in vitro and in vivo [121], which is regarded as pioneering research in EDT. The following studies all focused on combining certain substances with PtNP in order to achieve better antitumor effects than plain PtNP. Lu et al. incorporated glucose-oxidase (GOx) into porous platinum nanospheres; GOx can catalyze the oxidation of glucose to generate H_2_O_2_ in the tumor microenvironment, and the H_2_O_2_ is subsequently decomposed by the platinum nanospheres and generates O_2_ to facilitate the glucose consumption by GOx. The combination of GOx-mediated tumor starvation and EDT exhibited good antitumor effects both in vitro and in vivo [122]. Similarly, Chen et al. combined the glutamine antagonist 6-diazo-5-oxo-l-norleucine (DON) with PtNP in order to eliminate the anti-ROS glutathione [123]. Another study by Chen et al. incorporated Fe_3_O_4_ into PtNP to facilitate ROS generation as well as GSH depletion [124].

So far, most of the studies on EDT have focused on modifying the tumor microenvironment; future studies can lay more emphasis on targeted delivery or combinatorial therapies. In addition, clinical translation is encouraged, since Pt has been indicated to possess lower cytotoxicity and stronger stability in the physiological environment [122].

## 9. Conclusions and Future Perspectives

The recent development of various stimuli-responsive treatments and nanomaterials has attracted many researchers with different academic backgrounds. Some clinical studies have already indicated the potential and clinical efficacy of certain types of stimuli-responsive therapies such as photodynamic and photothermal therapies in head and neck squamous cell carcinoma treatment, while a large number of preclinical studies have provided us with various novel nanoparticle designs with better biocompatibility, better targeting effects, lower cytotoxicity and promising future clinical applications.

Photodynamic treatment has been used clinically for quite a long time and has been extensively studied both clinically and preclinically. Hence, the research progress of photodynamic treatment is far ahead of the remaining types of stimuli-responsive treatments. Thus, the recent progress in photodynamic nanoparticle designs such as tumor microenvironment modification, targeted delivery, more favorable biodegradability, boosted photodynamic effects and external stimuli-free design can all be referenced and applied in other less extensively studied therapies such as sonodynamic treatment, radiodynamic treatment, magnetodynamic treatment, etc.

Various preclinical studies especially on photodynamic and photothermal therapy have indicated that the stimuli-responsive therapies have shown a very good synergistic effect with chemotherapy, immunotherapy, and other types of stimuli-responsive therapy. Thus, combinatorial treatment should be paid more attention especially in those less extensively studied therapies such as sonodynamic, radiodynamic, magnetodynamic, electrodynamic and microwave dynamic therapy. Additionally, some agents with the characteristic of being excited by more than one type of external stimuli are natural candidates for combinatorial treatment.

More translational studies are also required since the clinical studies lag far behind the preclinical studies; the synthesized nanoparticles are getting more complex and more multifunctional, making their preclinical results better. Though the newer generation of stimuli-responsive nanoparticles is more potent in preclinical research, manufacturing complexities are hindering its clinical translation. In order to facilitate future clinical translation, efficacy, ease of use, inexpensiveness and ease of synthesis should be paid more attention to. More prompt clinical trials are encouraged when the safety of the agent can be ensured and the therapeutic effect is proven to be superior to the current treatment regimen. Considerable room exists for the preclinical and clinical expansion of various stimuli-responsive treatments with nanotechnology innovations and therapeutic strategy improvements.

## Data Availability

Not applicable.
